# The Effects of Aging on Clinical Vestibular Evaluations

**DOI:** 10.3389/fneur.2015.00205

**Published:** 2015-09-22

**Authors:** Maxime Maheu, Marie-Soleil Houde, Simon P. Landry, François Champoux

**Affiliations:** ^1^École d’orthophonie et d’audiologie, Université de Montréal, Montréal, QC, Canada; ^2^Centre de recherche interdisciplinaire en réadaptation du Montréal métropolitain (CRIR), Institut Raymond-Dewar (IRD), Montréal, QC, Canada

**Keywords:** VEMP, vHIT, caloric vestibular stimulation, vestibular function tests, aging

## Abstract

Balance disorders are common issues for aging populations due to the effects of normal aging on peripheral vestibular structures. These changes affect the results of vestibular function evaluations and make the interpretation of these results more difficult. The objective of this article is to review the current state of knowledge of clinically relevant vestibular measures. We will first focus on otolith function assessment methods cervical-VEMP (cVEMP) and ocular-VEMP (oVEMP), then the caloric and video-head impulse test (vHIT) methods for semicircular canals assessment. cVEMP and oVEMP are useful methods, though research on the effects of age for some parameters are still inconclusive. vHIT results are largely independent of age as compared to caloric stimulation and should therefore be preferred for the evaluation of the semicircular canals function.

## Introduction

With the accelerating aging of the global population, age-related health issues are becoming a growing concern (1). Increased risks of falls from loss of balance are among these health concerns (2) and are considered by the WHO as an important burden on both the health care system and health of the population (3). Vestibular information, which provides information related to static and dynamic position in space, is known to play a major role in balance (4). Better understanding of the effects of aging on the vestibular system can thus be beneficial in addressing risks of fall in an aging population.

The vestibular system is located in the inner ear and is composed of three semicircular canals (lateral, anterior, and posterior) that detect angular acceleration and two otolith organs (saccule and utricule) that detect gravity (5). Information from the saccule and the posterior semicircular canal projects to central vestibular pathways mostly through the inferior vestibular nerve. Information from the utricule, the lateral, and the anterior semicircular canals mostly projects through the superior vestibular nerve (5) and primarily reaches the vestibular nuclei (5).

The integrity of these vestibular structures is essential for normal balance. Investigations on the vestibular system and aging have revealed reductions in many vestibular structures, including otoconia in both otolith organs (6, 7), vestibular hair cells in the horizontal crista ampullaris (8), scarpa ganglion neurons (9–11), and vestibular nuclei neurons (12, 13).

The objective of this article is to review the current state of knowledge of clinically relevant vestibular measures part of a comprehensive assessment protocol. Indeed, with the aging population and the increasing data from scientific research, it is becoming crucial to take a critical look at the effects of aging on clinical test results to gain insight on best practices for clinical vestibular evaluation in an elderly population.

## Comprehensive Assessment of the Vestibular System

Healthcare professionals such as audiologists frequently perform vestibular testing. These evaluations are done to detect vestibular deficits in order to improve vestibular compensation and to reduce both dizziness and unsteadiness. A comprehensive clinical test battery should include appropriate techniques to assess the otolith organs and the semicircular canals.

The vestibular-evoked myogenic potential (VEMP) is an electrophysiological technique used to assess otolith function and can be evoked using different stimuli, such as sounds, vibrations, and electrical stimulations (14–17). Two responses can be evoked using VEMP: ocular-VEMP (*oVEMP*) and cervical-VEMP (*cVEMP*).

The cVEMP is an inhibitory response measured from the ipsilateral sternocleidomastoïd muscle and originates from the saccule (16, 18). The inhibition response is measured from the first positive peak occurring around 13 ms (p13 or P1) followed by a negative peak around 23 ms (n23 or N1).

The oVEMP is an excitatory response of the inferior oblique eye muscle contralateral to the stimulated ear and is thought to originate from the utricle (18, 19). The oVEMP response is composed of a negative peak occurring at around 11–12 ms (N1) and positive peak occurring at around 18 ms (P1) (20). These responses have been found to be robust indicators of vestibular system integrity and are independent from hearing abilities (21, 22).

Caloric stimulation is a commonly used clinical vestibular evaluation method that induces a movement of the endolymph within the horizontal semicircular canal by applying either warm or cold water or air to the external ear canal (23). Despite its wide clinical use, the mechanisms underlying caloric stimulation are contested [for more details see Ref. (24)].

The video-head impulse test (vHIT) is a more recent technique for the functional assessment of all six semicircular canals (25). This latest vestibular clinical assessment tool uses an infrared camera designed to track pupillary movement and a patient-worn gyroscope mounted on goggles (26). Gain, the vHIT’s output, is calculated by comparing eye and head velocity during fast head movements in each of the six semicircular canals’ planes (25, 26). vHIT can provide useful information in the assessment of semicircular canal function through the vestibulo-ocular reflex (VOR) (25, 27). Measurement of the vertical canal function is based on the 2D modified HIT technique (28). This method yields higher gains for the vertical canals than the 3D HIT coil technique. vHIT is highly sensitive to calibration error. This can lead to erroneous calculation of gain magnitude and make comparison between studies difficult.

## Effects of Aging and Vestibular Evaluation Techniques

### Evaluation of otolith function

The effects of normal aging on VEMP responses have been analyzed for different parameters, such as peak-to-peak amplitude [the difference between both components (microvolt)], latency [time of occurrence of both components (millisecond)], thresholds [lowest intensity of a stimuli to trigger a response], response rate [prevalence of a VEMP response (%)], and asymmetry ratio [difference in amplitude between individual ears].

#### cVEMP and Aging

One of the most widely observed effects of aging with cVEMP is a decrease in recorded amplitude (15, 19, 29–35). Indeed, this decrease has been reported to happen at a rate of 0.14 μV per decade (31) and is independent of the stimulus used (35). On the other hand, cVEMP thresholds have been observed to remain steady up to 50 years of age (15) and then progressively increase (15, 19, 34). This increase has been reported for click and tone burst stimulations (12). Though the majority of studies report no significant effect of age on cVEMP latencies (19, 31, 33, 35), few authors have observed age-related increased latency on the p13 (15, 34) and n23 (9), while other have reported age-related decreases to n23 (24). cVEMP response rates are widely reported to decrease with age. However, the age at which this happens is still contested. Some report a response rate of 100% for participants <65 years old followed by a rapid decline (32), while others have observed a progressive decline starting at 50 years old (15).

#### oVEMP and Aging

Numerous studies have reported a decrease in oVEMP amplitude related to age (19, 29, 31, 33, 35–37). More specifically, oVEMP amplitude has been reported to decrease by 2.9 μV per decade, independent of the stimulus used (19, 29, 31, 33, 36, 37). Normal aging has been reported to increase oVEMP thresholds (12). Most studies report significant increases in oVEMP latency with age (19, 31, 33, 36, 37). This increase has been reported to be significant after 60 years of age (36) and at a rate of 0.12 ms per decade (31). Interestingly, it has been suggested that this increase is only significant in men (33). To the best of our knowledge, only one study did not report any significant difference in oVEMP latencies between age groups (35). oVEMP response rate has been shown to decrease with normal aging (36, 37) but can be dependent on the method of stimulus presentation used (36). A significant reduction in response rate for participants over 60 years of age can be measured when stimuli are presented by bone conduction though no significant differences in response rate were found for galvanic stimulation.

### Evaluation of semicircular canal function

#### Caloric and Aging

Caloric stimulation output is based on the slow-phase movement of the eyes during caloric evoked nystagmus. Few recent researches have looked at the effects of aging on caloric stimulation. Investigations on the effects of normal aging on caloric response found a significant increase in response for middle-aged groups followed by a slow decline with increasing age (32, 38, 39). However, this difference was only noted for the warm irrigation (32, 39). Despite these results suggesting an effect of aging on caloric responses, some investigations have failed to uncover any age-related differences (40, 41).

#### vHIT and Aging

Recent advances in eye-tracking technology have led to the development of the vHIT. Numerous methods exist to calculate vHIT gain. The studies described in this review divided the area under the eye velocity curve by the area under the head velocity curve (42). In healthy adults, gain is typically between 0.8 and 1.2 (26). Investigations on the effects of aging on vHIT gain have only suggested a minor effect (26, 42–44). However, a higher head rotation velocity can elicit significant differences after 70 years old (45, 46). The decline in gain per decade has been suggested to be 0.012 (45) and significant gain differences have only been found beginning at 90 years of age (45). Till now, research on the effects of aging on vHIT has primarily investigated the effect of aging on the horizontal semicircular canals. To the best of our knowledge, only one study assessed the effect of age on vertical canals (42). The authors found that age did not influence vHIT response for anterior canals and gain only slightly decreased with age for the posterior canals.

## Discussion

Current clinical vestibular evaluation methods provide information on the integrity of the peripheral vestibular system. Understanding results from these evaluations in the context of normal aging is crucial to properly diagnosing vestibular disorders. Despite the well-known effects of aging on the peripheral vestibular system (6, 8), the effects of normal aging on vestibular evaluations, as highlighted by this review, is of yet a largely contested field (see Table 1). Understanding these effects has important clinical implications to help delineate various vestibular pathologies in an aging population.

**Table 1:** **Effects of aging on clinical vestibular evaluation techniques**.

Evaluation technique	Parameter	Effect of aging	Reference
cVEMP	Amplitude	Decrease	(15, 19, 29–35)
	Thresholds	Increase	(15, 19, 34)
	Latencies	Inconclusive	(15, 19, 31–35)
	Response rate	Inconclusive	(15, 32)
oVEMP	Amplitude	Decrease	(19, 29, 31, 33, 35–37)
	Thresholds	Increase	(19)
	Latencies	Increase^a^	(19, 31, 33, 36, 37)
	Response rate	Inconclusive	(36, 37)
Caloric	Slow-phase velocity	Inconclusive	(38–41, 47)
vHIT	Gain	No significant effect^b^	(32, 42–46)

*^a^Possible gender effect*.

*^b^Decrease at high rotary velocities*.

From the existing research, it is possible to propose guidelines for the use of clinical vestibular evaluations in an aging population. For instance, cVEMP is a useful tool to evaluate saccule function and the inferior vestibular nerve for the elderly when used with precaution. Reduced amplitude is reported with aging but these parameters should still be considered valuable, especially as amplitude asymmetry ratios are shown to remain stable with age (31, 35). Despite their inconsistencies in research, latencies can also be valuable complementary parameters. In fact, as most studies report no significant change in cVEMP latencies parameters, a change in latency should not be attributed to aging and should draw the attention of the clinician (19, 31, 33, 35). Taken together, these last two parameters can allow clinicians to identify vestibular pathologies using cVEMP without significant age-related interference.

Ocular-VEMP can be a useful evaluation method for utricule function and superior vestibular nerve integrity if it is used attentively. Similarly to cVEMP, amplitude remains a valid parameter for oVEMP evaluation as there is no significant asymmetry despite a strong decrease (31). Latencies must be considered with caution as multiple investigations have reported significant age-related increases (19, 31, 33, 36, 37). It has also been suggested that this increase in latency is exclusive to men (33). We therefore propose the use of clinician-established evaluation norms taking age and gender into account.

On the other hand, caloric testing should be used with precaution for an aging population. Till now, there is debate surrounding the mechanisms underlying caloric responses (47). Therefore, non-vestibular factors beyond normal aging could influence the results (40). The lack of unanimity for caloric responses in relation to age, combined with the debate surrounding the mechanisms underlying this method (47), are indicators that caloric testing still requires investigation before conclusive opinions on the effects of aging can be drawn.

We propose that vHIT is the preferable method to evaluate the vestibular canals in an aging population, although some precaution is needed regarding neck stiffness to avoid neck injuries. Results from vHIT appear to be largely independent of normal aging (45, 46) as only the posterior semicircular canal demonstrates a slight decrease in gain with increasing age (42). Therefore, semicircular canal function of older patients should be evaluated using vHIT. However, further studies are needed to measure the effect of aging for vHIT on the vertical semicircular canals.

## Conclusion

This review provided an overlook for the existing literature for the effects of aging on clinical vestibular evaluations. Of the most popular evaluation methods, vHIT is largely independent of age and should be preferred over caloric to evaluate semicircular function. This recommendation is due to a lack of understanding of the underlying mechanisms of caloric stimulation and the inconstant reported effects of aging. cVEMP and oVEMP are also useful methods, though it should be kept in mind that research on the effects of aging are still inconclusive for some parameters.

## Conflict of Interest Statement

The authors declare that the research was conducted in the absence of any commercial or financial relationships that could be construed as a potential conflict of interest.

## Funding

This work was supported by grants from the Fonds de Recherche du Québec – Santé (FRQS).
